# Lecithin Prevents Cortical Cytoskeleton Reorganization in Rat Soleus Muscle Fibers under Short-Term Gravitational Disuse

**DOI:** 10.1371/journal.pone.0153650

**Published:** 2016-04-13

**Authors:** Irina V. Ogneva, Nikolay S. Biryukov

**Affiliations:** 1 Department of Molecular and Cell Biomedicine, State Scientific Center of Russian Federation Institute of Biomedical Problems of the Russian Academy of Sciences, Moscow, Russia; 2 I.M. Sechenov First Moscow State Medical University, Moscow, Russia; 3 Moscow Institute of Physics and Technology (State University), Moscow region, Russia; University of California, Davis, UNITED STATES

## Abstract

The aim of this study was to prevent the cortical cytoskeleton reorganization of rat soleus muscle fibers under short-term gravitational disuse. Once a day, we injected the right soleus muscle with 0.5 ml lecithin at a concentration of 200 mg/ml and the left soleus muscle with a diluted solution in an equal volume for 3 days prior to the experiment. To simulate microgravity conditions in rats, an anti-orthostatic suspension was used according to the Ilyin-Novikov method modified by Morey-Holton et al. for 6 hours. The following groups of soleus muscle tissues were examined: «C», «C+L», «HS», and «HS+L». The transversal stiffness of rat soleus muscle fibers after 6 hours of suspension did not differ from that of the control group for the corresponding legs; there were no differences between the groups without lecithin «C» and «HS» or between the groups with lecithin «C+L» and «HS+L». However, lecithin treatment for three days resulted in an increase in cell stiffness; in the «C+L» group, cell stiffness was significantly higher by 22.7% (p < 0.05) compared with that of group «C». The mRNA content of genes encoding beta- and gamma-actin and beta-tubulin did not significantly differ before and after suspension in the corresponding groups. However, there was a significant increase in the mRNA content of these genes after lecithin treatment: the beta-actin and gamma-actin mRNA content in group «C+L» increased by 200% compared with that of group «C», and beta-tubulin increased by 100% (as well as the mRNA content of tubulin-binding proteins *Ckap5*, *Tcp1*, *Cct5* and *Cct7*). In addition, desmin mRNA content remained unchanged in all of the experimental groups. As a result of the lecithin injections, there was a redistribution of the mRNA content of genes encoding actin monomer- and filament-binding proteins in the direction of increasing actin polymerization and filament stability; the mRNA content of *Arpc3* and *Lcp1* increased by 3- and 5-fold, respectively, but the levels of *Tmod1* and *Svil* decreased by 2- and 5-fold, respectively. However, gravitational disuse did not result in changes in the mRNA content of *Arpc3*, *Tmod1*, *Svil* or *Lcp1*. Anti-orthostatic suspension for 6 hours resulted in a decrease in the mRNA content of alpha-actinin-4 (*Actn4*) and alpha-actinin-1 (*Actn1*) in group «HS» compared with that of group «C» by 25% and 30%, respectively, as well as a decrease and increase in the ACTN4 protein content in the membrane and cytoplasmic fractions, respectively. Lecithin injection resulted in an increase in the *Actn1* and *Actn4* mRNA content in group «C+L» by 1.5-fold and more than 2-fold, respectively, compared with the levels in group «C». Moreover, in group «HS+L», the mRNA content did not change in these genes compared with the levels in group «C+L», and the ACTN4 protein content in the membrane and cytoplasmic fractions also remained unchanged. Thus, lecithin prevented the reduction of *Actn1* and *Actn4* mRNA and the migration of ACTN4 from the cortical cytoskeleton to the cytoplasm.

## Introduction

Currently, one of the most medically significant challenges to long-term space missions, including ones to other planets, such as Mars, is manifested muscle atrophy, which does not allow the performance of necessary work after landing on the surface of a body in space. Moreover, despite various countermeasures, cosmonauts and astronauts still require a number of recovery procedures after the long-term orbital spaceflight and their subsequent return to Earth, which is not possible for flights to other planets.

Exposure to microgravity conditions for long periods of time has been shown to result in significant weight loss and atrophic changes in the soleus muscle [1,2,3]. Moreover, a decrease in functional capacity has been reported for the entire muscle [4,5] and its isolated fibers [6]. In rodents, anti-orthostatic suspension is accompanied by similar effects on skeletal muscle [7].

It is clear that a decrease in the functional potential of postural muscles when exposed to microgravity conditions occurs when the integrity of basic structural and functional muscle tissue units, such as a muscle fiber (single cell) is disrupted. Nevertheless, mechanisms of the mechanoreception of different cells, including muscle cells, still remain unclear.

The extracellular matrix and membrane proteins [8,9], mechanosensitive and/or other ion channels [10,11,12], structures of the submembrane (cortical) cytoskeleton [13], and intracellular structures [14,15,16], in particular, could all act as mechanosensors. However, nearly all potential mechanisms of primary mechanotransduction are dependent on the condition of the submembrane cortical cytoskeleton, and the structural integrity of which is dependent on the content of actin and actin- binding proteins, and determines the mechanical properties of various types of cells, which is ultimately reflected in the stiffness of cells.

Currently, there are data demonstrating that alpha-actinin-4 dissociates from the cortical cytoskeleton into the cytoplasm in the soleus muscle cells at the earliest stages (6 hours) of hindlimb suspension [17]. Furthermore, subsequently, there are alterations in the mRNA content of the alpha-actinin-4 and decrease in gene expression of cytochrome *c* (one of the basic in cell respiration) [18]. However, we can suggest that such dynamics may be typical not only for the alpha-actinin-4, but also for other actin-binding proteins like as proteins of the Arp2/3 complexes, tropomodulin, supervillin, L-plastin. That is why we analyzed their gene expression.

The dissociation of actin-binding proteins from the cytoskeleton results in a decrease in its stiffness. Alterations in stiffness of the cortical cytoskeleton due to changes in the actin filaments can activate some signaling pathways in different cell types [19,20,21,22,23,24].

However, in addition to changes in the external mechanical tension, changes in the cholesterol content in the membrane can also result in a reorganization of the cortical actin cytoskeleton. There are data that indicate that an association with lipid micro domains of plasmatic membrane (rafts) rich in cholesterol can be an essential factor defining the activity of the integrated membrane proteins, including ionic channels [25,26,27,28,29,30]. The rupture of the structure and consistency of the rafts caused by a decrease in the level of membrane cholesterol interfered with cellular functions, including reorganization of the actin network [28,31]. Furthermore, it has been shown that a partial extraction of membrane cholesterol using methyl-beta cyclodextrin inhibited the mechanosensitive activation of channels in K562 cells [32,33]. In cells with a lowered content of cholesterol, there was an observed increase in the threshold of activation and a decrease in the probability of the open state of channels. Furthermore, measurements of mechanosensitive flows in various conditions and complementary data of fluorescent microscopic analyses indicated that suppression of the activity of mechanosensitive channels is mediated by the reorganization of actin, which was initiated, according to several studies, by a rupture of raft consistency, resulting from decreased levels of membrane cholesterol [32,33]. But as well as cholesterol content changes, treatment cells by unsaturated lipids (for example, lecithin) may result in cortical cytoskeleton reorganization.

In this investigation, we examined the role of cortical cytoskeleton stiffness (directly associated with non-muscle actin isoforms and actin-binding proteins) in cell mechanosensitivity. We found changes in the stiffness of the soleus muscle fibers due to lecithin injections, which consequently prevented alpha-actinin-4 migration from the membrane to cytoplasm under anti-orthostatic suspension conditions.

## Materials and Methods

The experiments were performed using soleus muscle tissue obtained from Wistar rat (n = 14 animals). Control animals (n = 7) were housed under vivarium conditions and received standard vivarium food for rodents and water, which were provided *ad libitum*. The control animals were 203 ± 10 g. To simulate the microgravity conditions in rodents, antiorthostatic suspension was performed according to the Ilyin-Novikov standard method modified by Morey-Holton et al. [7] for 6 hours during day hours (from 9 a.m. to 3 p.m.). The suspended animals (n = 7) were 210 ± 10 g (there was no difference in the mass between the control and experimental groups of animals). The animals during suspension were housed in the same light and temperature conditions as the control animals and received standard vivarium food for rodents and water *ad libitum*. We monitored the suspended animals every 15 minutes during whole suspension period. In our previous study we showed by measurements of the corticosterone level that there were no significant increase stress level during 6 hours of suspension [17].

Dry lecithin was diluted in 0.9% NaCl + 1% C_2_H_5_OH diluted in water. The mice were injected once a day with this solution in the right soleus muscle with 0.5 ml lecithin at a concentration of 200 mg/ml. In the left soleus muscle, an equal volume of diluted solution was injected. All injections started for 3 days prior to the suspension. The main idea was to increase cortical cytoskeleton stiffness for preventing ACTN4 migration to cytoplasm at the start of suspension. We monitored the animals twice a day, and no animals became ill or died prior to the experimental endpoint.

**Fig 1:**
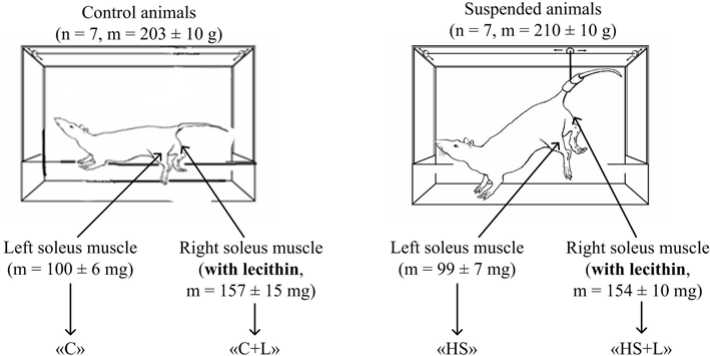
Experimental design.

Animals were euthanized with an overdose of Avertin, and the following groups of soleus muscle tissues were obtained: «C», «C+L», «HS», «HS+L» (Fig 1). The soleus muscle mass remained unchanged during the period of anti-orthostatic suspension between the corresponding legs. For the left leg (injection of diluted solution without lecithin), there were 100 ± 6 mg for group «C» and 99 ± 7 mg for group «HS». For the right leg (injection of the lecithin), there were 157 ± 15 mg for group «C+L» and 154 ± 10 mg for group «HS+L». Thus, there was a significant increase (p < 0.05) in the soleus muscle mass after three days of lecithin injections compared with animals receiving injections of the diluted injections.

All procedures with animals were approved by the biomedical ethics committee of the State Research Center of Russia Institute of Biomedical Problems of the Russian Academy of Sciences.

### Transverse stiffness measurements of single cells using atomic force microscopy

Soleus muscles were excised transversely from one tendon to another and treated according to the technique previously described by Stevens et al. [34].

Prior to the experiments, samples were stored at -20°C in a buffer containing equal parts of relaxation solution R (20 mM MOPS, 170 mM of potassium propionate, 2.5 mM of magnesium acetate, 5 mM of K_2_EGTA and 2.5 mM of ATP) and glycerol.

On the day of the experiment, the samples were transferred to solution R where isolated glycerynized muscle fibers were isolated.

To measure the transversal stiffness, the obtained fibers were fixed on the bottom of the liquid cell of the atomic force microscope (NT-MDT, Russia), and their tips were attached with special Fluka shellac wax-free glue (Sigma, Germany). The measurements were performed using contact mode with an indentation depth of 150 nm according to a technique that has been previously described in detail [35].

The obtained results were processed using MatLab 6.5 software.

### Reverse transcription and real-time PCR (RT-PCR) for mRNA quantification

To determine the mRNA content of the genes encoding the cytoskeletal proteins, total RNA was isolated from the frozen samples of rat soleus muscle, using the RNeasy Micro Kit (Qiagen, Germany) according to the manufacturer’s manual. Reverse transcription was performed using d(T)_15_ as the primers and 500 ng of RNA. To evaluate the expression rates of the studied genes, real-time PCR (RT-PCR) was performed using specific primers selected with Primer3Plus software (Table 1). Melting curves were performed to ensure the fidelity of the PCR product. The 2(-Delta DeltaC(T)) method [36] was used to determine the fold difference.

**Table 1:** RT-PCR primers and products.

Gene	Direction	Primer sequence (5'…3')	Product size, bp
***Actb* (beta-actin)**	Forward/ Reverse	gctgcgttttacaccctttc/ gtttgctccaaccaactgct	218
***Actg* (gamma-actin)**	Forward/ Reverse	ctggtggatctctgtgagca/ tcaggagggaagaaaccaga	184
***Arpc3* (actin related protein 2/3 complex, subunit 3)**	Forward/ Reverse	acaggaggatgaaacgatgc/ tcacaaagcaagtccaccac	125
***Tmod1* (tropomodulin 1)**	Forward/ Reverse	actggaaaacgagctggatg/ gcttgcttttccaagtggtc	138
***Svil* (supervillin)**	Forward/ Reverse	tcggaaaccaagacgctatc/ accactggcaattctgaacc	132
***Lcp1* (lymphocyte cytosolic protein 1)**	Forward/ Reverse	gaagggatcgtcaaacttgc/ tatggttctgaggggattgg	130
***Actn1* (alpha-actinin 1)**	Forward/ Reverse	ggtcagcagcaacctcctc/ tctttctccaccttctctcca	167
***Actn4* (alpha-actinin 4)**	Forward/ Reverse	accctgaacagactcccttg/ gatcgacaagcctccatctc	168
***Des* (desmin)**	Forward/ Reverse	gtgaagatggccttggatgt/ cgggtctcaatggtcttgat	182
***Tubb2b* (tubulin, beta 2B class IIb)**	Forward/ Reverse	ggcagcaagaagctaacgag/ cgaacacgaagttgtctggc	302
***Ckap5* (cytoskeleton associated protein 5)**	Forward/ Reverse	tgctgaaagacctgatgcac/ ccaaaaccttcaccaccaag	117
***Tcp1* (t-complex 1)**	Forward/ Reverse	gctgcaatcaccatacttcg/ ctccgtgtttgtcgtctttg	73
***Cct5* (chaperonin containing Tcp1, subunit 5—epsilon)**	Forward/ Reverse	caaacgggctggataagatg/ tcctgggatttggacagttc	136
***Cct7* (chaperonin containing Tcp1, subunit 7—eta)**	Forward/ Reverse	tgtgaccgtgaagaagcaag/ gcatcatcacagcatcaacc	137
***Cycs* (cytochrome c)**	Forward/ Reverse	ccaaatctccacggtctgtt/ tctgccctttctcccttctt	190
***Gapdh* (glyceraldehyde 3-phosphate dehydrogenase)**	Forward/ Reverse	acccagaagactgtggatgg/ acacattgggggtaggaaca	172

### Determination of the protein content using western-blotting analyses

To determine the protein content, soleus muscle samples were frozen in liquid nitrogen. This method has been previously described by Vitorino et al. [37] and was used to prepare tissue extracts to obtain the membranous and cytoplasmic fractions of proteins. After sample preparation spectrophotometry was used to measure the total protein concentration in each sample. Denaturizing polyacrylamide gel electrophoresis was performed using the Laemmli technique and the Bio-Rad system (USA). On the basis of the measured concentrations of total protein content, equal amounts of protein were added to each well. Transfer onto nitrocellulose membrane was performed as described by Towbin et al. [38]. We used total protein staining of the membrane as a normalization control. For each protein, specific monoclonal primary antibodies based on mouse immunoglobulins were used (Santa Cruz Biotechnology, Inc.) in dilutions recommended by the manufacturer: 1:100 –for alpha-actinin-1 (ACTN-1, MW = 100 kDa); 1:100 –for alpha-actinin-4 (ACTN-4, MW = 105 kDa); 1:100 –for cytochrome *c* (CYCS, MW = 15 kDa). For secondary antibodies, biotinylated goat antibodies against mouse IgG (Santa Cruz Biotechnology Inc.) diluted 1:5000 were used. Next, all membranes were treated with streptavidin conjugated with horseradish peroxidase (Sigma, Germany) diluted 1:5000. Protein bands were identified using 3,3’-diaminobenzidine (Merck, USA). ImageJ software was used to quantify the western blot bands.

### Statistical analysis

The results obtained during these experiments were statistically processed with ANOVA, using the post hoc *t*-test with a confidence level p < 0.05 to evaluate the significance of differences between groups. These data were represented as the M ± SE, where M is the mean and SE is the standard error.

## Results

### Transverse stiffness of single muscle fibers

**Fig 2:**
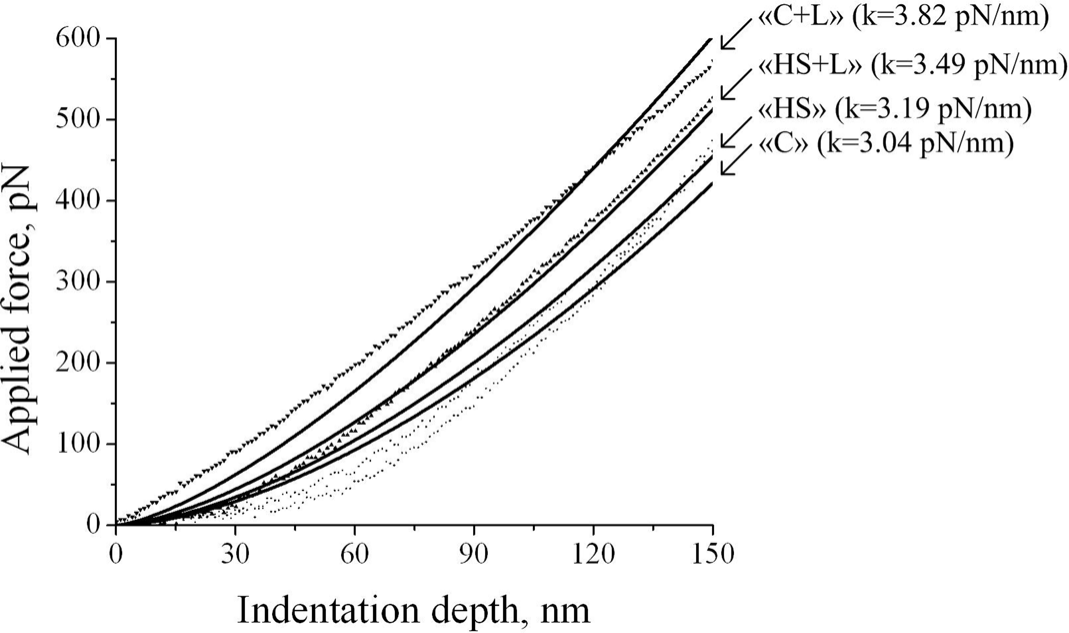
Typical force curves obtained using transversal stiffness measurements of rat soleus muscle fibers under 6 hours short-term (6 hours) anti-orthostatic suspension with and without lecithin treatment (see Material and methods section).

The transversal stiffness of rat soleus muscle fibers after 6 hours of suspension did not differ from the control group level for the corresponding legs: there were no differences between groups without lecithin «C» (3.08 ± 0.10 pN/nm) and «HS» (3.17 ± 0.08 pN/nm) as well as between groups with lecithin «C+L» (3.78 ± 0.11 pN/nm) and «HS+L» (3.53 ± 0.09 pN/nm) (Fig 2). However, in group «C+L», cell stiffness was significantly higher by 22.7% (p < 0.05) than in group «C». Thus, lecithin treatment for three days resulted in an increase in cell stiffness.

### Content of mRNA in genes encoding main cytoskeletal and metabolic proteins

**Fig 3:**
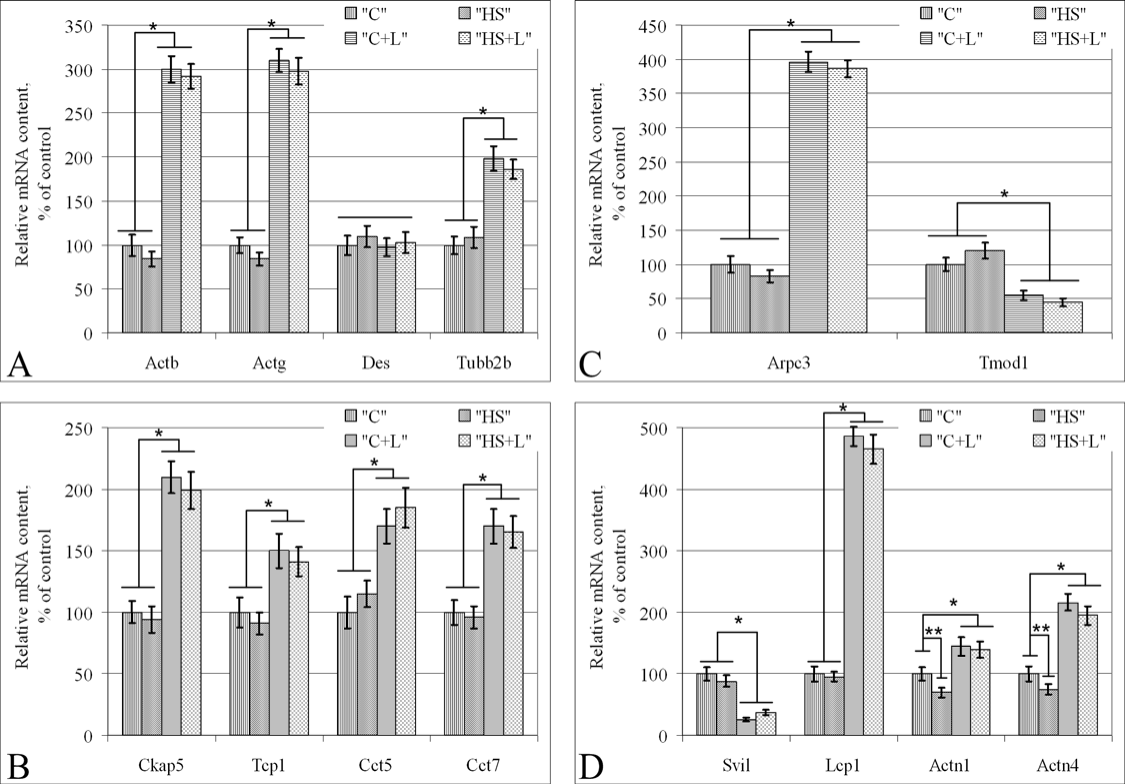
Expression level of genes encoding cytoskeletal proteins in the soleus muscle fibers of rats after short-term gravitational unloading. (A) Main cytoskeletal proteins: microfilaments (*Actb*, *Actg*), microtubules (*Tubb2b*), intermediate filaments (*Des*). (B) Microtubules- and tubulin-binding proteins. (C) Actin monomers-binding proteins. (D) Actin filaments-binding proteins. Hereafter: a horizontal line determines equal means; *–p < 0.05 means of group «C+L» compared to group «C» in this figure, **–p < 0.05 means of group «HS» compared to group «C».

The mRNA content of genes encoding beta- and gamma-actin and beta-tubulin did not significantly differ before and after suspension in the corresponding groups (between «C» and «HS», between «C+L» and «HS+L») (Fig 3A). However, there was a significant increase in the mRNA content of these genes after lecithin treatment; the beta-actin and gamma-actin mRNA content in group «C+L» was increased by 200% compared with that of group «C», and beta-tubulin increased by 100%. In addition, the desmin mRNA content remained unchanged in all experimental groups.

The mRNA content of the genes encoding tubulin- and microtubules-binding proteins was the same as the mRNA content of beta-tubulin; there were no changes after hindlimb unloading. However, after lecithin treatment, the mRNA content of *Ckap5*, *Tcp1*, *Cct5* and *Cct7* in group «C+L» increased by 110% (p < 0.05), 50% (p < 0.05), 70% (p < 0.05) and 70% (p < 0.05), respectively, compared with the levels in group «C» (Fig 3B).

The mRNA content of genes encoding actin monomer-binding proteins subunit 3 of actin related protein 2/3 complex (*Arpc3*) and tropomodulin 1 (*Tmod1*) did not change from control levels after gravitational disuse (Fig 3C). Nevertheless, lecithin treatment resulted in a redistribution of its expression level; *Arpc3* mRNA content in group «C+L» was increased by 296% (p < 0.05) compared with group «C», but the mRNA content of *Tmod1* decreased by 45% (p < 0.05).

Similar results were obtained for the actin filament-binding proteins supervillin (*Svil*) and lymphocyte cytosolic protein 1 (*Lcp1*); there were no changes in the mRNA content after anti-orthostatic suspension and redistribution after lecithin treatment (Fig 3D). *Svil* mRNA content decreased approximately 5-fold (by 74%, p < 0.05) in group «C+L» compared to group «C», but the mRNA content of *Lcp1* increased approximately 5 times (by 386%, p < 0.05).

The mRNA content of the alpha-actinin-1 gene (*Actn1*) and alpha-actinin-4 gene (*Actn4*) in group «HS» (after 6 hours of anti-orthostatic suspension) decreased by 30% (p < 0.05) and by 25% (p < 0.05), respectively, compared with group «C» (Fig 3D). In group «C+L», *Actn1* and *Actn4* mRNA content were significantly higher compared with group «C» by 45% (p < 0.05) and by 116% (p < 0.05), respectively. Moreover, their contents remained unchanged after 6 hours of disuse (in group «HS+L» compared to group «C+L»).

**Fig 4:**
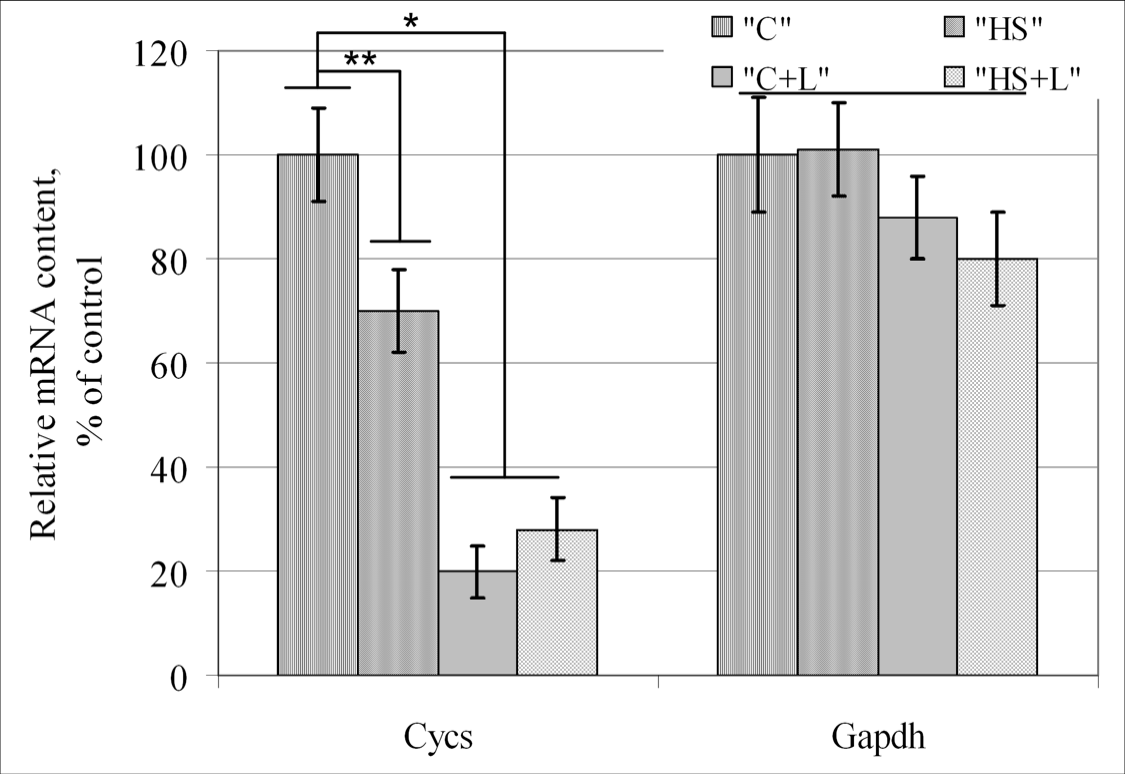
Expression levels of genes encoding metabolic proteins in the soleus muscle fibers of rats after short-term gravitational unloading: cytochrome *c* (*Cycs*) and glyceraldehyde 3-phosphate dehydrogenase (*Gapdh*).

The mRNA content of the gene encoding glyceraldehyde 3-phosphate dehydrogenase (*Gapdh*) remained constant in all groups (Fig 4). In addition, the mRNA content of cytochrome *c* (*Cycs*) in group «HS» decreased by 30% (p < 0.05) compared with group «C». In the control group with lecithin treatment, «C+L», the *Cycs* mRNA content significantly decreased by 80% (p < 0.05) compared with group «C». However, there were no significant changes between groups «C+L» and «HS+L».

### Relative protein content of alpha-actinin-1, alpha-actinin-4 and cytochrome c

**Fig 5:**
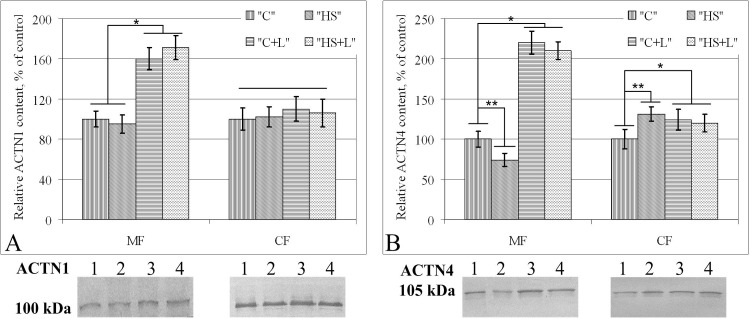
Relative protein content in the membrane protein fraction (MF) and cytoplasmic protein fraction (CF) of soleus muscle fibers of rats after short-term gravitational unloading. (A) Alpha-actinin-1 (ACTN1, MW = 100 kDa) content. (B) Alpha-actinin-4 (ACTN4, MW = 105 kDa) content. On the typical Western blot images: 1 –group «C», 2 –group «HS», 3 –group «C+L», 4 –group «HS+L».

The alpha-actinin-1 (ACTN1) content in the membrane fraction did not change after 6 hours of suspension; however, in groups treated with lecithin, the content was significantly higher compared with the content in the groups without lecithin treatment; in group «C+L», the ACTN1 content increased by 60% (p < 0.05) compared with the level of group «C» (Fig 5A). However, in the cytoplasmic fraction of proteins, the relative content of ACTN1 remained unchanged in all groups.

In addition, the ACTN4 content changed after 6 hours of suspension in group «HS» compared with group «C»; the content was reduced by 26% (p < 0.05) in the membrane fraction and increased by 31% (p < 0.05) in the cytoplasmic fraction (Fig 5B). In group «C+L», the relative content of ACTN4 increased by 110% (p < 0.05) in the membrane fraction compared with that of group «C», and no significant changes were observed in the cytoplasmic fraction. After 6 hours of suspension with lecithin treatment, there were no significant changes in the ACTN4 relative content (in group «HS+L» compared with group «C+L»).

**Fig 6:**
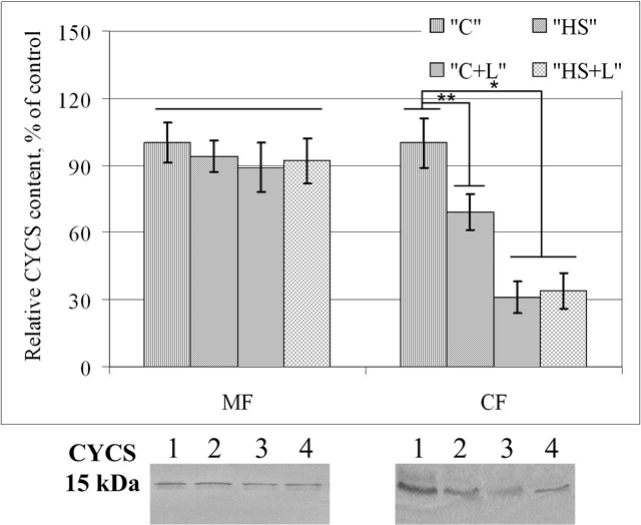
Relative cytochrome *c* (CYCS, MW = 15 kDa) content in the membrane-mitochondrial protein fraction (MF) and cytoplasmic protein fraction (CF) of soleus muscle fibers of rats after short-term gravitational unloading. On typical Western blot images: 1 –group «C», 2 –group «HS», 3 –group «C+L», 4 –group «HS+L».

The cytochrome *c* relative content in the membrane-mitochondrial fraction remained unchanged in all experimental groups (Fig 6). In the membrane fraction, there were significant decreases of 31% (p < 0.05), 69% (p < 0.05) and 66% (p < 0.05) in groups «HS», «C+L» and «HS+L», respectively, compared with the level in group «C». However, there were no significant changes between groups «C+L» and «HS+L».

## Discussion

The deep space exploration problem remains unresolved, particularly with regard to its medical impacts. The absence of effective countermeasures of the muscle system is mainly due to the lack of studies on the interaction mechanisms between cells and gravitational fields.

We previously proposed that the cortical cytoskeleton plays a key role in cell mechanosensitivity. In case of an increase external mechanical field alpha-actinin-1 dissociated from cortical cytoskeleton, in case of decrease–alpha-actinin-4 migrated from cortical cytoskeleton to cytoplasm As a result there could be activation of different signaling pathways and consequent cellular responses [17,39,40].

For this reason, we aimed to prevent alpha-actinin-4 migration from the cortical cytoskeleton into the rat soleus fibers cytoplasm from the membrane during anti-orthostatic suspension. We performed gene expression screening analysis of representative genes from major cytoskeletal protein families to identify alternative potential primary mechanotransductors under gravitational unloading in addition to alpha-actinin-4.

We assumed that alpha-actinin-4 migration from the membrane fraction to the cytoplasm in rat soleus muscle fibers occurs due to cortical cytoskeleton deformation [41]. Thus, if we increased cortical cytoskeleton stiffness, would a reduction in deformation under gravitational unloading occur?

It is well known that changes in cholesterol content in the membrane can lead to the reorganization of the cortical actin cytoskeleton due to changes in anchoring sites [25,26,27,28,29,30,31]. Introduction of unsaturated lipids, such as lecithin, into the cell membrane may result in similar effects. Unsaturated phospholipids are known to display protective properties in all cell types [42].

### General parameters: muscle mass, transversal cell stiffness and mRNA of main cytoskeletal proteins content

The obtained results indicate that 3-day lecithin-treated muscle mass («C+L» and «HS+L» groups) increased 1.5 times compared with those of the «C» and «HS» groups and remained stable for 6 hours of gravitational unloading. Moreover, the transversal stiffness of the membrane with the cortical cytoskeleton under gravitational unloading did not change. Moreover, in groups «C+L» and «HS+L», transversal stiffness was by 22% higher compared with group «C» and «HS».

Since stiffness is primarily dependent on the condition of the cortical cytoskeleton [43,44,45,46], we proposed that the gene expression levels of the main cytoskeletal proteins should also increase as a result of lecithin injection. Indeed, the mRNA content of genes encoding actin non-muscle isoforms (*Actb* and *Actg*) was 3 times greater in the lecithin-treated groups. Furthermore, the quantity of microtubule mRNA components, such as *Tubb2b*, was also 2 times higher in lecithin-treated groups. In addition, the amount of desmin (protein forming intermediate filaments) mRNA did not differ from control levels after lecithin introduction. Importantly, anti-orthostatic suspension did not result in changes in gene expression in the corresponding groups: there were no difference between groups «C+L» and «HS+L» and between groups «C» and «HS».

### mRNA content of tubulin-binding proteins

Naturally, an increase in the mRNA content of microtubule components is followed by an increase in the mRNA content of tubulin-binding proteins, such as *Ckap5*, *Tcp1*, *Cct5* and *Cct7*, which demonstrated a 1.5–2 times increase after lecithin injection. CKAP5 (XMAP215)–XMAP215/Dis1 family of cytoskeletal proteins bind microtubules together and to the membrane forming tubulin cytoskeleton. Moreover, XMAP215 associates with the barbed ends of microtubules, preventing its degradation [47], and contributes to the cleavage spindle shaping process in somatic cells [48]. TCP1, CCT5 and CCT7 proteins are components of chaperonins-containing polypeptide t-complex 1, which contains 8 subunits (CCT 1–8) [49] and is involved in the folding of cytoplasmic proteins, particularly actin and tubulin. Newly synthesized alpha- and beta-tubulin subunits assemble into heterodimers in the presence of Mg and ATP with the help of the TCP1 complex [50]. A decrease in the content of CCT subunits combined with chaperone activity level, depending on the cell cycle stage, particularly in M-phase, is restored in S-phase [51]. Moreover, the TCP1 complex plays a role in cyclin E synthesis, promoting cell movement from G_1_ to S phase through the cell cycle [52]. However, anti-orthostatic suspension did not result in changes in the tubulin-binding content.

### mRNA content of actin-binding proteins: *Arpc3*, *Tmod1*, *Svil* and *Lcp1*

However, the mRNA content of actin-binding proteins showed improved dynamics when the actin mRNA content increases.

ARP2/3 represents the macromolecular protein complex consisting of ARP2, ARP3 and five additional proteins [53] and plays a role in the nucleation and *de novo* assembly of actin filaments [54]. It has been proposed that ARP2 and ARP3 bind together such that they become similar to a stable actin dimer with opened barbed ends [55], and the presence of ARP3 is essential [56]. Nucleation may occur on the cytoplasmic membrane, where ARP2/3 is localized [57]. It activates with the help of minor G-proteins, such as Scar (cAMP receptor suppressor), WASP (Wiskott-Aldrich syndrome protein) and WAVE (WASP-verprolin homologs) [58,59]. Importantly, gravitational unloading does not affect the Arpc3 mRNA content, whereas lecithin injection increases this parameter more than four-fold.

Furthermore, lecithin injection results in a two-fold decrease in tropomodulin mRNA content (*Tmod1*), which blocks actin filaments elongation [60], but remains stable during anti-orthostatic suspension.

A similar redistribution occurs as a result of lecithin injection in the mRNA content of genes encoding proteins that bind to actin polymers. Thus, there was a 5-fold reduction in the supervillin mRNA content in lecithin-treated muscle fibers. Supervillin is a peripheral membrane protein that binds myosin II and F-actin in every cell, reconstructing the actin cytoskeleton and negatively regulating stress fibrils, focal adhesive contacts and cell substrate adhesion [61]. Moreover, the mRNA amount of L-plastin is increased 5 times after lecithin injection. L-plastin is an actin-binding family protein, is considered to be highly conserved in eukaryotes and is present in various types of cells [62]. It may either bind to F-actin in the cortical cytoskeleton or play a role as the three-dimensional actin network main organizer in the cell depending on the phosphorylation level [63].

Thus, lecithin injection in rat soleus muscles induces a redistribution of gene expression towards actin polymerization enhancement and actin filaments stabilization. These genes encode proteins and bind to actin monomers and actin filaments. However, gravitational unloading does not affect *Arpc3*, *Tmod1*, *Svil* and *Lcp1* mRNA content.

### mRNA and relative protein content of actin-binding proteins (alpha-actinin-1 and alpha-actinin4) and cytochrome c

Six hours of anti-orthostatic suspension results in a decrease in alpha-actinin-4 and alpha-actinin-1 mRNA content in the «HS» group compared with the «C» group by 25% and 30%, respectively, which is consistent with our previous data [17]. Lecithin injection resulted in an increase in *Actn1* and *Actn4* mRNA content by 1.5- and 2-fold, respectively. In addition, the mRNA content of these genes did not vary in the «HS+L» group compared with group «C+L». Thus, lecithin prevented a decrease in the *Actn1* and *Actn4* mRNA content after 6 hours of gravitational unloading.

However, we previously showed that the alpha-actinin-1 protein content in the membrane and cytoplasmic fraction in rat soleus muscles after 6 hours anti-orthostatic suspension remained stable, whereas alpha-actinin-4 content decreased in the membrane fraction and increased in the cytoplasm [17]. In this study, the similar results were obtained. For this reason, we analyzed the content of these proteins after lecithin injection. In the «C+L» group, the ACTN1 and ACTN4 contents were increased by 1.6- and 2.1-fold, respectively, compared with the «C» group. However, in the «HS+L», the relative content of these proteins did not differ from that in the «C+L» group, i.e., lecithin prevented ACTN4 migration from the membrane to the cytoplasmic fraction in rat soleus muscle fibers under anti-orthostatic suspension.

ACTN4 is known to interact with nuclear proteins. Thus, alpha-actinin-4 interacts with NF-Y to either attract chromatin remodeling complexes or to signal for the activation of target genes and transcription complexes [64]. Moreover, alpha-actinin-4 may independently penetrate into the nucleus and bind to the promoter region, which contains a GCTGCCGCAC-(N4-20)-GGSCGYGGG sequence, similar to the cytochrome *c* promoter [65].

For this reason, we analyzed the gene expression level and protein content of cytochrome *c* in both the mitochondrial membrane and cytoplasmic fractions. *Cycs* mRNA content was reduced by 30% in «HS» compared with the «C» group, and lecithin injection prevented this reduction: there was no difference between the *Cycs* mRNA content in «C+L» and «HS+L». However, lecithin injection resulted in a 5-fold decrease of *Cycs* mRNA in «C+L» compared with the «C» group.

In addition, the cytochrome *c* content in the membrane-mitochondrial fraction remained0020030stable after both anti-orthostatic suspension and after lecithin injection. However, there was a 30% decrease in the cytochrome *c* content in the cytoplasmic fraction after suspension and a three-fold decrease as a result of lecithin injection. However, lecithin prevents cytochrome *c* reduction in the cytoplasmic fraction after 6 hours of gravitational unloading. Importantly, there was a decrease in cytochrome *c* after injection of other polyunsaturated phosphatdylcholines during rat alcoholization, and these effects are decreased following ethanol-induced increases in hepatocyte apoptosis due to caspase-3 content and activity reduction [66].

## Conclusions

In this study, we aimed to identify gene candidates that are targets of changes in the external mechanical field. There were no changes in the mRNA content of genes encoding cytoskeletal proteins, except the actin-binding proteins alpha-actinin-1 and alpha-actinin-4, in soleus muscle fibers after 6 hours of rat gravitational disuse. Moreover, changes in the protein content (migration from the membrane to the cytoplasmic fraction of proteins) were observed only for alpha-actinin-4. These data on alpha-actinin-1 and alpha-actinin-4 are consistent with our previous data.

Thus, we aimed to prevent this ACTN4 migration. We used lecithin to strengthen the cortical cytoskeleton by injection into the soleus muscle. Consequently, we observed an increase in the muscle mass, single fiber transversal stiffness and mRNA content of genes encoding major cytoskeletal proteins. We prevented the migration of ACTN4 from the membrane fraction to the cytoplasmic fraction of proteins. Because ACTN4 can migrate to the nucleus and regulate the expression of different genes, including cytochrome *c*, we measured the cytochrome *c* protein and mRNA content. Lecithin prevented the decrease in cytochrome *c* content under gravitational unloading; however, the cytochrome *c* content in the cytoplasmic fraction was decreased. This result may not be negative because increases in the cytochrome *c* content in the cytoplasm result in apoptosis.

Thus, lecithin treatment of the rat soleus muscle before 6 hours of gravitational unloading prevented the reorganization of the cortical cytoskeleton and may be protective of the cytochrome *c* content in the cytoplasmic fraction of proteins.

Although we analyzed the mRNA content of major genes encoding tubulin- and actin-binding proteins, one limitation of this study was that we only examined a limited number of genes. Moreover, the effect of lecithin on cell respiration could be contradictory. On one hand, it is negative due to the decrease in mRNA and protein content under lecithin treatment. On the other hand, it is positive because there was no change in the cytochrome *c* content in mitochondria, and there was a decrease in the cytoplasmic fraction. The latter effect can reduce the level of apoptosis.
